# Service-Oriented Model Encapsulation and Selection Method for Complex System Simulation Based on Cloud Architecture

**DOI:** 10.3390/e21090891

**Published:** 2019-09-14

**Authors:** Siqi Xiong, Feng Zhu, Yiping Yao, Wenjie Tang, Yuhao Xiao

**Affiliations:** College of Systems Engineering, National University of Defense Technology, Changsha 410073, China; siqi@mail.ustc.edu.cn (S.X.); ypyao@nudt.edu.cn (Y.Y.); tangwenjie@nudt.edu.cn (W.T.); xiaoyuhao19@nudt.edu.cn (Y.X.)

**Keywords:** complex system simulation, cloud computing architecture, service-oriented modeling, semantic search framework, QoS-based service selection

## Abstract

With the rise in cloud computing architecture, the development of service-oriented simulation models has gradually become a prominent topic in the field of complex system simulation. In order to support the distributed sharing of the simulation models with large computational requirements and to select the optimal service model to construct complex system simulation applications, this paper proposes a service-oriented model encapsulation and selection method. This method encapsulates models into shared simulation services, supports the distributed scheduling of model services in the network, and designs a semantic search framework which can support users in searching models according to model correlation. An optimization selection algorithm based on quality of service (QoS) is proposed to support users in customizing the weights of QoS indices and obtaining the ordered candidate model set by weighted comparison. The experimental results showed that the parallel operation of service models can effectively improve the execution efficiency of complex system simulation applications, and the performance was increased by 19.76% compared with that of scatter distribution strategy. The QoS weighted model selection method based on semantic search can support the effective search and selection of simulation models in the cloud environment according to the user’s preferences.

## 1. Introduction

The continuous evolution of complex systems (e.g., social systems, ecosystems, and war systems) has had a tremendous impact on people’s daily life and social development. Due to the limitation of existing theoretical analysis methods and the difficulty of experimental analysis methods in some real-world complex systems (e.g., geological changes, nuclear explosions, economic growth [1], and ecosystem evolution), complex system simulation technology has gradually become an attractive approach for the research on complex systems and their complexity [2].

Complex system simulation applications often contain a large number of simulation model entities, and there are complex interactions between these entities, also the entities and the external environment. Such system simulations usually have a large computational load [3]. With the increase in scale and complexity of complex system simulation applications, there are increasingly requirements for the composite mode of simulation models, the computational capabilities of simulation architectures, and the execution efficiency of simulation applications. The popularity of cloud computing technology provides a new approach, platform architecture, and efficient computing power for the research and development of complex system simulations. Simulation users can use the computing resources in the cloud environment on demand at different terminals and invoke the simulation model services stored in the cloud center to assemble complex system simulation applications. Therefore, the development of service-oriented simulation models has gradually become a prominent topic in the field of complex system simulation [4]. Service-oriented technology is mainly directed at models with large computational requirements, such as an electromagnetic environment calculation model, a ballistic path planning model, a radar detection model, and so forth. These models are expected to be provided outward as a shared service. Then, their integration and code porting could be eliminated, and the construction efficiency of complex system simulation applications and the utilization of related models could be improved. The interoperability between heterogeneous simulation models and the distributed collaborative calculation of simulation models on multiple computing nodes could be realized, which could improve the execution efficiency of simulation applications. Therefore, it is necessary to carry out the research on how to construct and select the service-oriented complex simulation models based on cloud computing environment.

To make the complex system simulation model into a shared service in cloud, firstly, models with large computational requirements need to be encapsulated into simulation services in the cloud environment and be parallel processed in the execution of complex system simulation applications under a cloud-based simulation model service framework. Because the simulation model services released and stored in the cloud center have differences in attributes, functions, and quality of service (QoS), it is necessary to find and select the appropriate simulation model that accurately meets the user’s requirements in terms of function and can provide high QoS for building complex system simulation applications [5]. Reusable model development (RUM) specification [6] cannot support network communication between a simulation model and a simulation engine under the cloud architecture. Ontology web language [7] (OWL)-based simulation model search methods lack the mechanism to search simulation models through the correlations between models. Also, current simulation model optimization selection algorithms lack the induction for QoS [8] attributes of simulation models in the cloud environment and cannot provide a selection mechanism that satisfies the user’s preference for a model’s QoS.

In order to solve the abovementioned problems in the existing studies, this paper proposes a service-oriented model encapsulation and selection method for complex system simulation based on cloud architecture. The novelty and contribution of this method includes that it designs a cloud-service-oriented reusable model development (C-RUM) specification to encapsulate the simulation model into a shareable simulation service in the cloud, and then devises a cloud-based simulation model service framework, which solves the problem of network communication in the former RUM specification. This method also uses a knowledge graph [9] to describe the simulation model services and establishes a model semantic search framework in the constructed model description knowledge graph, which supports users in setting correlations between models to obtain the required model. A QoS weighted-based optimization selection algorithm is also proposed, which can select the optimal simulation model that satisfies the user’s preference for QoS according to a weighted comparison of QoS indices.

The organization of this paper is as follows: Section 2 discusses related works. Section 3 introduces the C-RUM specification and the cloud-based simulation model service framework. Section 4 introduces the selection method of simulation models based on semantic search. Section 5 describes a case study of the service-oriented model encapsulation and selection method. Section 6 is the summary and the outlook for future work.

## 2. Related Works

### 2.1. High-Level Architecture (HLA)-Based Simulation Model Development Specification

The basic idea of HLA is to use an object-oriented method to design, develop, and implement object models of different levels and granularities and to obtain high-level interoperability and reusability of simulation models and simulation systems. The object model template (OMT) is a standardized description of the properties of simulation models and their interaction formats, but it is not a standard for establishing the object model. With the development of complex system simulation, there are higher requirements for the efficiency, flexibility, and openness of simulation model development. HLA-based simulation model development specification gradually exposes some problems in the application process, such as efficiency, ease of use, fault tolerance, dynamic compatibility, and so forth [10].

### 2.2. RUM Specification

In order to realize the interapplication and interplatform reuse of simulation models and the rapid development of simulation applications, many researchers have proposed reusable and composable development specifications and methods for simulation models. Lee et al. applied the product line engineering concept to the development of simulation model components [11]. Feng et al. proposed a reusable component model development approach for parallel and distributed simulation, requiring that the simulation model have self-contained features; that is, the model can be packaged and released independently, without relying on other models, and is separate from the simulation engine [6]. Jianbo and Yiping proposed a reusable component model framework (RCMF) model development tool called SuKit, which can be used to regenerate models and guide model integration [12]. 

A patent for RUM specification [13] was proposed by Yiping and Feng and revised in 2017, which has been widely used. RUM specification encapsulates the simulation model into a separate service entity, and the model and the outside world can only interact through the “service interface”. RUM specification enables local reuse and composition of simulation models, realizing invocation and communication of simulation models by passing local parameters. However, in the cloud environment, the user terminal and the cloud server are connected by the network, and RUM specification does not support communication between the simulation model and the specific simulation engine framework in the network. Therefore, the simulation model developed by RUM specification cannot be provided as a shared service released and stored in the cloud environment.

### 2.3. OWL-Based Simulation Model Search Method

Ontologies in the Semantic Web can describe simulation models at the semantic level. Web service ontology description language (OWL-S) was designed to make the Web service an entity which computers can understand based on the description of ontology. OWL-S describes Web services in three aspects: (1) service profile, (2) service model, and (3) service grounding [14,15]. Ontology can improve the accuracy of simulation model search by describing simulation models based on semantics [16]. In order to support the composite modeling of complex system simulation applications, some experts have carried out research on simulation service description methods based on semantics and have proposed description ontologies of simulation model resources (e.g., OWL-SS [17] and OWL-SM [18]). At present, OWL-based simulation model description methods generally lack descriptions of the characteristics of simulation models in the cloud environment [19] and lack expression of the correlations between simulation models, which are not effective enough to support users in searching and selecting relevant models conveniently through the correlations between models in the cloud environment.

### 2.4. QoS-Based Simulation Model Selection Method

Similar to Web services, QoS is a key factor in choosing simulation models that are stored in the cloud environment as a service [20]. At present, many researchers have defined suitable QoS indices for simulation services and have proposed model selection mechanisms based on QoS [21]. However, current descriptions of simulation models lack the induction of QoS attributes of simulation models in the cloud environment [22]. Current selection algorithms lack a selection mechanism that can select models in the cloud environment according to users’ preferences for QoS indices and thus cannot meet users’ specific QoS requirements when constructing complex system simulation applications.

In summary, current HLA-based simulation model development specification and RUM specification cannot support simulation models as a shared service to be invoked and operated on cloud architecture by developers. Existing OWL-based model search methods and QoS-based model selection methods cannot support users in searching for relevant models through the correlations between models and selecting simulation models that meet their QoS requirements to construct complex system simulation applications in the cloud environment.

## 3. C-RUM Specification and Cloud-Based Simulation Model Service Framework

### 3.1. C-RUM Specification

Complex system simulation applications usually contain some simulation models which have a large computational load. The operation of such a model requires an immense amount of computing resources, making other simulation models in the same process fall into a long queue, thus delaying the advancement of the simulation timing and reducing the execution efficiency of the simulation application. If such simulation models are encapsulated in the form of shared services and are distributed and stored in the cloud environment, the construction efficiency of complex system simulation applications and the utilization of related models could be improved. Cloud computing resources can be used to realize interoperability between heterogeneous simulation models and distributed collaborative computing of simulation models on multiple computing nodes, so as to improve the execution efficiency of simulation applications.

**Figure 1:**
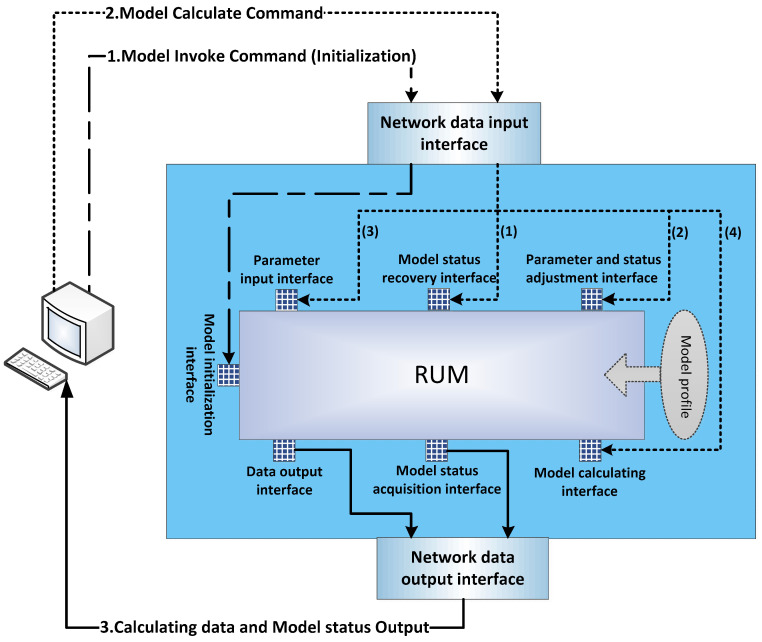
Cloud-service-oriented reusable model development (C-RUM) specification and its execution flow.

The RUM specification can implement local invocation and communication of simulation models. However, in the cloud architecture, the cloud server where the simulation service model is located and the user terminal are interconnected through the network. The RUM specification does not support communication between the simulation model and the specific simulation engine framework on the network. This paper proposes C-RUM specification by transforming the RUM specification. The purpose is to invoke the simulation model encapsulated by C-RUM specification as a form of shared service in the cloud architecture, to make the model service transmit the data through the network protocol to interact with the simulation engine framework, and to implement distributed collaborative computing and heterogeneous execution of simulation applications. The original RUM specification specifies seven standard (service) interfaces for the simulation model to interact with the outside world—model initialization, parameter input, model status recovery, parameter and status adjustment, data output, model status acquisition, and model calculating interfaces—to provide seven standard operations, as shown in Figure 1.

In the cloud architecture, the service interfaces in the original RUM specification cannot identify or parse the network data. Therefore, the C-RUM specification defines the network data input interface and network data output interface. These two interfaces are used to encapsulate the seven service interfaces in order to implement data conversion between the network and the original interfaces. According to the execution flow of the simulation model under the original RUM specification [6], the C-RUM specification divides the network interaction into three types: model invoke command, model calculate command, and calculating data and model status output, as shown in Figure 1. The detailed function of the two new interfaces and the network interaction of the C-RUM-encapsulated simulation model is discussed below.

Network data input interface. This interface is mainly used for parsing network data transmitted based on the socket network transmission protocol. The purpose of the parsing is to obtain the target standard service interface of the network data packet and convert the input data into the specified standard data format of the corresponding service interface. Finally, the parsed instructions or parameters are passed to the target standard service interface. The model invoke and calculate commands need to be analyzed by the network data input interface.

Model invoke command: When the simulation system of the user terminal needs to be initialized, the terminal will send a model invoke command to the simulation model in the cloud server, and the distributed invocation system in the cloud server will mount the simulation service model into a process. Then, the model initialization command and related parameters are passed to the model initialization interface through the network data input interface to complete the initialization operation of the simulation service model.

Model calculate command: When the simulation system in the user terminal needs the simulation service model to calculate, the model calculate command will be sent to the simulation model. After the command is parsed by the network data input interface, the model will (1) first recover its status through the model status recovery interface, (2) then check whether there is a working parameter adjustment instruction to adjust the working parameters and status, (3) then set the input data through the parameter input interface, and (4) finally start the simulation model calculating operation through the model calculating interface.

Network data output interface. This interface is used to encapsulate the data output from the simulation model after calculation and the status information of the simulation model. That includes indicating the standard interface source of the data and the destination address of the transmission and converting them into the format for the socket network communication protocol. Before output to the network, the calculating result and model status need to be encapsulated by the network data output interface.

Calculating result and model status output: After the simulation service model finishes its calculation, the network data output interface will obtain the data after calculating from the simulation model output interface and acquire the model status from the simulation model status acquisition interface and encapsulate the data into a socket communication protocol package. The package will be forwarded to the simulation system in the corresponding user terminal through the distributed architecture in the cloud environment.

### 3.2. Cloud-Based Simulation Model Service Framework

**Figure 2:**
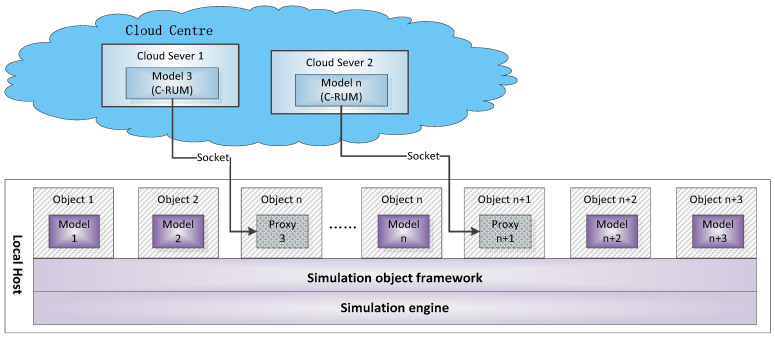
Cloud-based simulation model service framework.

Most of the current complex simulation system application frameworks need to integrate or migrate simulation models into specific simulation platforms, making it difficult to separate the models from the platform. For different simulation platforms, the operation mechanism of the simulation engine is quite different, and it is not easy to bind the service simulation model of the cloud center to a specific simulation platform. In order to invoke the service-oriented simulation model in the cloud architecture without relying on the simulation platform, running under any simulation engine framework [23,24], this paper proposes a cloud-based simulation model service framework, as shown in Figure 2.

The complex system simulation application consists of a large number of simulation object instances, and there are complex interactions and collaborative calculations between the instances. The simulation object framework is built on a specific simulation engine, and the simulation object instances are defined by the simulation object framework (including the declarations of these simulation object instances, their roles in the simulation application, the interaction between them, etc.). Each object instance is implemented by a specific simulation model. The local simulation model can be directly mounted or integrated into the object framework, while the simulation model service stored in the cloud environment relies on the communication with its proxy model in the corresponding object framework. The proxy model does not have the specific function of the simulation service model; it only takes the place of the simulation service model in the entire simulation object framework, defining the interaction relationship with other models. When the simulation application needs to interact with the simulation service model in the cloud server or obtain its status, the simulation object framework will accept and transmit the corresponding parameters and data through the socket communication between the proxy model and the simulation service model. The cloud-based simulation model service framework is applicable to the invocation and operation of the C-RUM-encapsulated simulation model in the cloud environment and does not depend on a specific simulation platform.

## 4. Simulation Model Selection Method Based on Semantic Search in Cloud Environment 

### 4.1. Semantic Search Framework

The traditional Web services description language (WSDL)-based [25] simulation model search mechanism uses keyword matching to find simulation model description texts with the same keywords. A knowledge graph uses a more expressive way to describe simulation models by semantic description, and a search method based on a knowledge graph can find simulation models at the semantic level through the link relations between data and things [26]. Compared with ontology description language, a knowledge graph stores resource description framework (RDF) [27] triples in the graph database directly, which means the correlations between simulation models can be described in a simple and intuitive way in the form of graphs.

**Figure 3:**
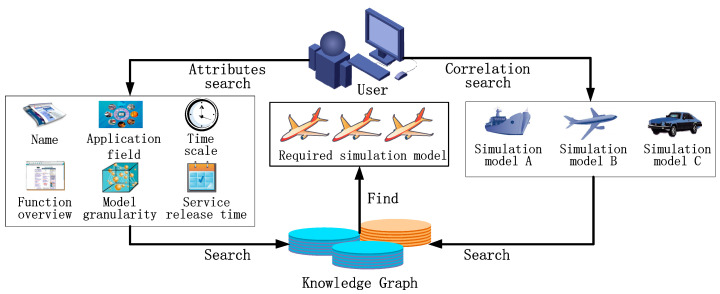
Simulation model semantic search framework.

In this study, a description method of cloud simulation model resources based on a knowledge graph [28] was used to describe simulation models, which describe the characteristics of cloud simulation models and their QoS indices. Then, a simulation model semantic search framework was proposed based on the simulation model description knowledge graph. This search framework provides two patterns for simulation model search: (1) users can associate the required simulation model by attribute information such as the name, domain, type, time scale, and model granularity of the simulation model; or (2) users can search for the required simulation model by the correlations between models. According to the search conditions input by the user, simulation models that meet the search conditions can be found in the knowledge graph stored in the graph database, as shown in Figure 3.

Under the search framework proposed in this paper, users input model attribute requirements as semantic search conditions stored in the array Attributes_conditions [M]. Each item of the array corresponds to 1 to M attribute requirements of the simulation model. The user can input one or more attribute requirements (e.g., model name, domain, category, time scale, model granularity, etc.) as semantic search conditions to search for simulation models that meet the requirements of these attributes. The user can also input the required association model and specific association relationships (e.g., command relationship, equipment-carrying relationship, etc.) as semantic search conditions stored in the two variables Correlated model and Relationship, respectively, to search for simulation models that have a certain relationship with the correlated model. The input of correlated models is necessary in this search pattern. Algorithm 1 shows the semantic search algorithm.

Data_Base represents a knowledge graph database that stores simulation model description information and correlation relationships. model ≮ α indicates that the simulation model does not satisfy the attribute requirement α by the judgment method of fuzzy search combined with synonym expansion. relationship (Correlated model, model) indicates the correlation between correlated model and present model. Relationship ≰ β indicates that the specified association relationship does not satisfy the correlation between correlated model and present model by means of fuzzy search combined with synonym expansion. push_into_list (model, Ω) indicates adding the simulation model into the model initial set Ω. 

**Algorithm 1** Semantic_Search**Input:***Attributes_conditions* [*M*], the vector for storing model attribute requirements;   *Correlated model*; *Relationship*, the relationship with correlated model;**Output:**Ω, simulation model initial set;1: Boolean flag1←true, flag2←true;2: **if** (*Attributes_search_conditions* ≠ *null*)||(*Relationship_search_conditions* ≠ *null*) then3:  **for each**
model∈Data_Base
**do //** Loop traversal of the simulation model4:    **for**
i←0
**to**
*M*
**do //** Loop traversal of model attribute requirement condition5:     **if**
model≮Attributes_search_conditions [i]
**then**
flag1←false;6:    **end for**7:    **if**
relationship (Correlation model, model)≠ NULL
8:     **if**
Relationship≰ relationship (Correlation model, model)        **then**
flag2←false;9:     **else**
flag2←false;
10:    **if** (*flag1* & *flag2*) **then**
*push_into_list (model*, Ω);11:  **end for**12: **end if**13: **return**
Ω


### 4.2. QoS Weighted-Based Simulation Model Selection Method in Cloud Environment

**Figure 4:**
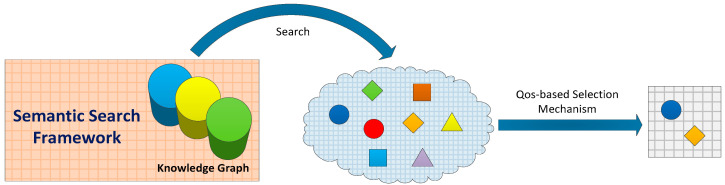
Search and selection process of simulation model.

The simulation model that the user needs to use has to not only meet the requirements of its function but also have high QoS to reach the quality requirements of building complex system simulation applications. The simulation models obtained under the semantic search framework proposed in Section 4.1. are not unique, and they have similar functions and attributes, but they differ in terms of QoS. In order to build higher-quality complex system simulation applications, after the initial set that meets the search conditions is acquired under the semantic search framework, it is necessary to order that set through a QoS-based selection mechanism and select the optimal simulation models from the ordered candidate set, as shown in Figure 4.

The QoS weighted-based simulation model selection mechanism proposed in this paper can support users in customizing QoS index weights and selecting the simulation model that satisfies their QoS preference from the initial set according to the weighted comparison of QoS indices. The specific method is discussed below.

Definition of QoS indices. Referring to the QoS indices of Web services and considering the uniqueness of the simulation model as a kind of special Web service [21,29], the QoS indices of the simulation model in the cloud environment can be summarized as follows:

1. Model performance (Q_M_) is determined by the computation of the model. A simulation model with more computations has lower model performance.
2. Communication capability (Q_C_) reflects the communication capability of the link between the user’s terminal node and the cloud server.
3. Availability (Q_A_) indicates the probability that the simulation model can be called and used. It is defined by the mean time between failures and the mean time to repair.
4. Reliability (Q_R_) is defined by the execution success rate of the service, which refers to the probability of obtaining the correct response to the user’s requirements within the maximum expected time range.
5. Security (Q_S_) is measured by the data management capability of a model service, which mainly depends on the user’s historical experience. Terminal users should be given a [0, 10] range to score the service (regarding the confidentiality, integrality, realness, etc., of data) after using it. Then, the value of Q_S_ is the average score; with the increase and accumulation of evaluations, this value becomes reliable.

QoS weighted-based selection algorithm. The above five attributes (Q_M_, Q_C_, Q_A_, Q_R_, and Q_S_) are all positive metrics; that is, the higher the value, the higher the quality. In order to eliminate the gap between the different QoS indices, we used the following formula [22] to limit their values to the range of [0, 1]:

(1) $$V\left(Q_{i}^{k}\right)=\frac{maxQ_{i}^{k}-Q_{i}^{k}}{maxQ_{i}^{k}-minQ_{i}^{k}}.$$

These five QoS indices are assigned numbers 1–5. $Q_{i}^{k}$ indicates the value of the *i*th QoS index of the *k*th model in the candidate set, $maxQ_{i}^{k}$ and $minQ_{i}^{k}$ indicate the maximum and minimum values, respectively, that the QoS index may reach, and $V\left(Q_{i}^{k}\right)$ indicates the value after standardization of this QoS index.

After entering the search condition under the search framework, the simulation user also needs to provide a QoS preference, which is expressed by a weight vector as the following formula:

(2) $$W=(w_{i}\;,1\leq i\leq 5,\sum w_{i}=1).$$

That is, the percentage each QoS index should be accounted for. According to the weight vector given by the user, the total QoS index of the *k*th model in the candidate set is

(3) $$\;Q^{k}=\overset{5}{\underset{i=1}{\sum }}w_{i}\leftarrow V\left(Q_{i}^{k}\right).$$

The model that meets the user’s search conditions under the semantic search framework will be added to the initial set. According to the weight vector representing the QoS preference provided by the user, the target QoS index Q of each model in the initial set is obtained by the above formulas. Finally, the candidate set of simulation models ordered by Q will be provided to the user for selection. Algorithm 2 shows the QoS weighted-based model selection process.

**Algorithm 2** QoS Weighted-Based_Selection**Input:** W, Simulation model QoS index weight vector;   Ω, Simulation model initial set (from Algorithm 1);**Output:**Φ, Ordered model candidate set;1: **if**
Ω≠null
**then**2:  **for each**
model∈Ω
**do        /**/ Loop traversal of model initial set3:    **for each**
i←0 to *5*
**do       //** Loop through 5 QoS indices4:     $V\left(Q_{i}\right)\;\leftarrow \frac{maxQ_{i}-Q_{i}}{maxQ_{i}-minQ_{i}}$    // Calculate the standard value of the QoS index5:     $Q\leftarrow \;\overset{5}{\underset{i=1}{\sum }}w_{i}\cdot V\left(Q_{i}\right)$  // Calculate the target QoS value of the simulation model6:     *push_into_list*(<model,*Q*>, Φ)  // Insert the binary <model, Q> into the set Φ7:    **end for**8:   **end for**9:  *rank_list_by* (Φ,Q)   // Sort the elements in Φ by Q10: **end if**11: **return**
Φ


## 5. Case Study: Airport Operation Control System Simulation

An airport operation control system simulation is mainly used to simulate the control and arrangement of an airport control center in different dispatching strategies. By simulating a period in the real world, simulation results of airliners’ punctuality rates and average delay times can be obtained. This complex system simulation provides an effective research method for the scheduling and control of airliners in airports. The airport operation control simulation system mainly includes airliner, airport runway, and air traffic control center (ATC) models. The airliner model records the delay time and has three statuses: taking off, landing, and waiting. The airport runway model records the idling and queuing status of runway. The ATC model needs to do many complex calculations based on relevant strategy, queue waiting of runways, and delay time of airliners, and then schedule the relevant airliners to wait on specified runways. Therefore, it takes much more time to calculate than the other two models. 

The abovementioned airport operation control system simulation was used as an experimental case to analyze the efficiency and practicability of the service-oriented model encapsulation and selection method for complex system simulation based on cloud architecture proposed in this paper. The simulation platform used in the case study was SUPE, and all experiments were run on two computing nodes with a Linux (centos7) operating system. Each node was equipped with a 3.40 GHz Intel (R) Core (TM) i7-6700 quad core CPU processor. Docker (version 1.13.0) container technology was used as a virtualization method to build a two-node cloud architecture, in which the distributed operation of the airport operation control simulation system was implemented. In the experiment, a simulation time was set up corresponding to the physical time of 10 min to study the actual system of 1 week (7 days), so each simulation promoted the logical simulation time of 1008. The time that was measured in the test was the execution time when the simulation application finished the 1008 simulation steps (logical simulation time). Each piece of experimental data in the analysis chart was the average value after 10 test runs. The experimental configuration is shown in Table 1.

### 5.1. Performance Evaluation

#### 5.1.1. Cloud-Architecture-Based Distributed Simulation (CDS)

In order to test the performance of the CDS (which refers to the architecture, the computing nodes of which communicate by a cloud architecture network), this experiment tested the execution time of the airport operation control simulation application under three operation modes: serial simulation on a single process in a single node (S-1P), traditional distributed simulation (TDS, which refers to the architecture, the computing nodes of which communicate by local connection) on two processes (TDS-2P, per process per node), and CDS on two processes (CDS-2P, per process per node) based on the above experimental parameter settings. TDS-2P and CDS-2P used the scatter distribution method (each type of simulation model was distributed to each process in turn), and each process ran in one node.

**Figure 5:**
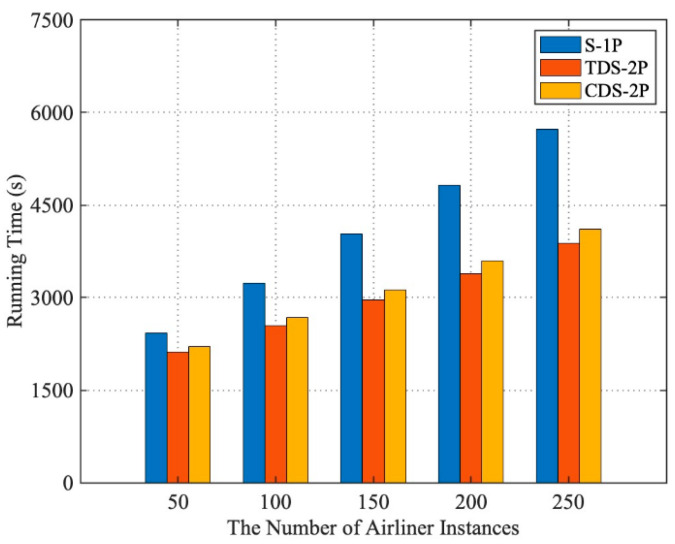
Running times of serial simulation on a single process in a single node (S-1P), traditional distributed simulation on two processes (TDS-2P), and cloud-architecture-based distribution on two processes (CDS-2P).

As shown in Figure 5, when the number of airliner instances was 50, 100, 150, 200, and 250, compared with the running time of the simulation application using the S-1P operation mode, both the CDS and TDS could reduce the running time of the simulation application and improve the execution efficiency. As the number of airliner instances increased, because the model distribution mode was scatter, the amount of computation assigned to each process would get closer to being equal. So, the running time of TDS-2P and CDS-2P would get closer to half that of S-1P. Compared with TDS, the performance of the CDS lost an average of 5.37% in five sets of experiments. This is because in the cloud architecture, Docker container technology uses virtualization to isolate interprocess resources. In CDS, processes at different nodes need to communicate through virtual addresses, which leads to higher communication latency than TDS. However, cloud computing has the advantages of computing resources being used on demand and service models being shareable, which would balance such performance loss. Therefore, it is feasible to use CDS architecture to run complex system simulations.

#### 5.1.2. Model Servitization

**Figure 6:**
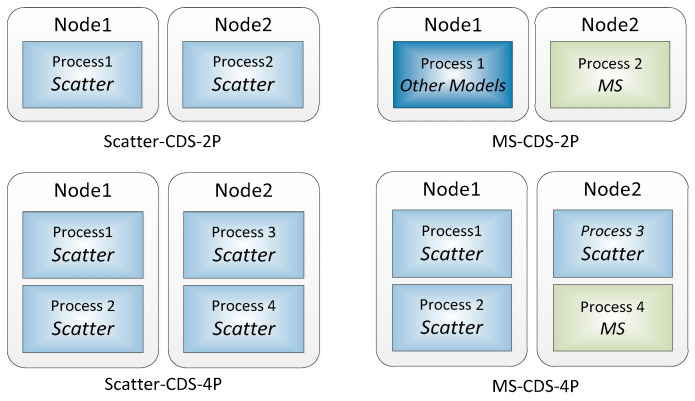
Operation and distribution modes.

This experiment packaged the simulation models with large computational requirements into a shareable simulation service through the C-RUM specification. The service model ran in parallel on a single process of a cloud server node, participating in the execution of a complex system simulation application in the cloud-based simulation model service framework (model servitization, MS). In order to study its performance, the case study encapsulated the ATC model, which has a greater number of calculations than the other models, into a service model based on the C-RUM specification. Then, we tested the effect of using scatter and MS distribution methods under CDS (Scatter-CDS and MS-CDS) on simulation execution time. The specific operation and distribution modes are shown in Figure 6. The experiment used two-process (per process per node) and four-process (two processes per node) parallel simulation to test the performance of Scatter-CDS and MS-CDS (Scatter-CDS-2P, MS-CDS-2P, Scatter-CDS-4P, and MS-CDS-4P). The MS distribution method operated the service model separately in one process, and the remaining models were distributed to the rest of the processes using scatter distribution.

**Figure 7:**
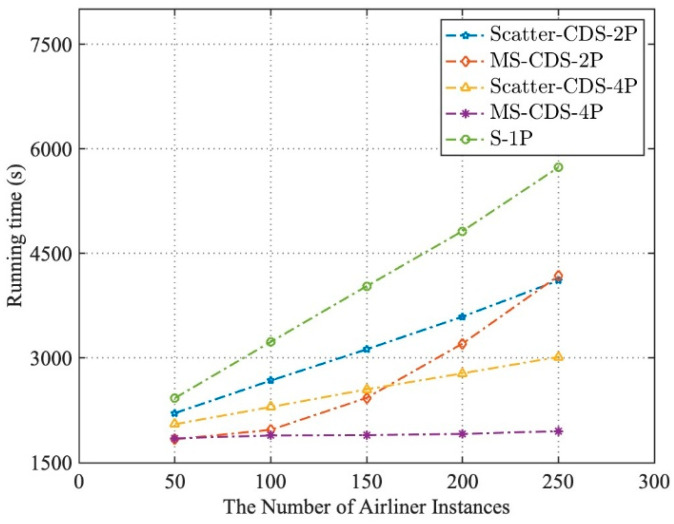
Running times of S-1P, Scatter-CDS-2P, model servitization (MS)-CDS-2P, Scatter-CDS-4P, and MS-CDS-4P.

**Figure 8:**
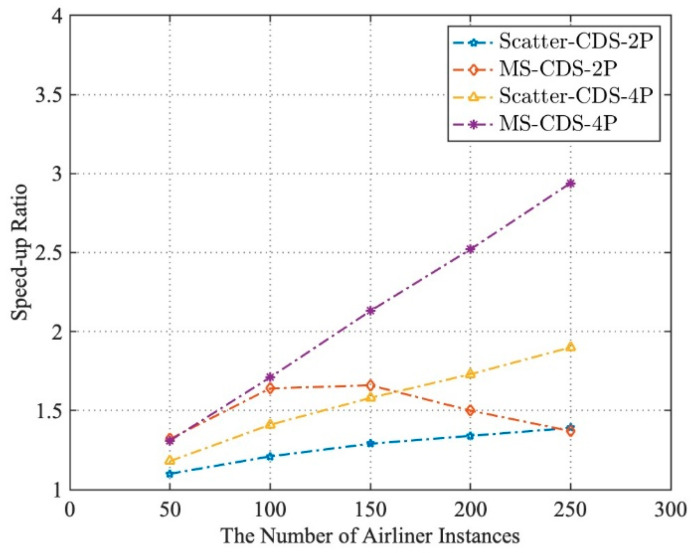
Speed-up ratios of S-1P, Scatter-CDS-2P, MS-CDS-2P, Scatter-CDS-4P, and MS-CDS-4P.

The results of the test are shown in Figure 7 and Figure 8, compared with the execution time of the simulation application by S-1P operation mode: (1) In the two-process parallel operation mode, when the number of airliner instances was less than 150, MS-CDS-2P was better able to reduce the execution time of simulation applications than Scatter-CDS-2P and had a higher running time speed-up ratio. However, when the number of airliner instances exceeded 150, the computation load was more unbalanced on two computing nodes, and the speed-up ratio of MS-CDS-2P gradually decreased and became even lower than that of Scatter-CDS-2P. (2) In the four-process parallel operation mode, MS-CDS-4P was better able to reduce the execution time of the simulation application and had a higher speed-up ratio (execution performance) than Scatter-CDS-4P when instantiating the number of airliners from 50 to 250. When the number of airliners was 250, the execution performance of MS-CDS-4P improved by 35.28% compared with Scatter-CDS-4P.

Through the experimental results and analysis, we found that packaging models with large computational loads into a shareable service, on the one hand, can provide support for quickly constructing the simulation system in the form of service combination. On the other hand, it can effectively improve the execution efficiency of the simulation system. Also, when the total calculation of the remaining models is gradually increased, the degree of parallelism should be increased to ensure load balancing, so as to maximize the effect of MS on increasing the execution efficiency of simulation applications.

#### 5.1.3. Simulation Model Selection Method Based on Semantic Search

In order to prove the practicability of the simulation model selection method based on semantic search proposed in this paper, five kinds of ATC service models with different QoS attribute characteristics were constructed by C-RUM specification. A simulation model description method based on a knowledge graph [28] was used to describe the simulation models of the airport operation control system simulation. The description information was added to the database that stored the model description knowledge graph (a knowledge graph that contained the description information of a large number of different models in various fields). Algorithm 1 was implemented by Cypher query language [30], and the semantic search framework was built in the model description knowledge graph database to find the required models. Then, based on Algorithm 2, according to different QoS index weight vectors, the simulation model candidate set with optimization order could be obtained for users to choose.

Under the simulation model semantic search framework, the five ATC service models (ATC-A, ATC-B, ATC-C, ATC-D, and ATC-E) could be accurately found by correlation with the airliner or runway model or by the attributes of the ATC model. These five models were added to the initial model set, and then based on the QoS index values of the five simulation models and QoS index weight vector, the target QoS value Q of each model could be obtained. The simulation model candidate set that was obtained by sorting Q was available for users to select. The experiment assumed that the user wants to select the service model that can optimize the execution efficiency of the simulation application. Directed at two operation modes, two QoS index weight vectors for different experimental methods were provided to select simulation models. The effectiveness of the semantic search framework and the QoS weighted-based model selection method was verified by running and testing the performance of the simulation application that was assembled by the selected ATC models 

(1) When the entire simulation system is running on a single node, the external network communication capability Qc does not affect the execution efficiency of the simulation application. The model performance Q_M_ dominates the effect (the other three QoS indices may have little effect on the execution efficiency), so the QoS index weight was set to W = (0.7, 0, 0.1, 0.1, 0.1), and the specific selection process is shown in Table 2.

**Figure 9:**
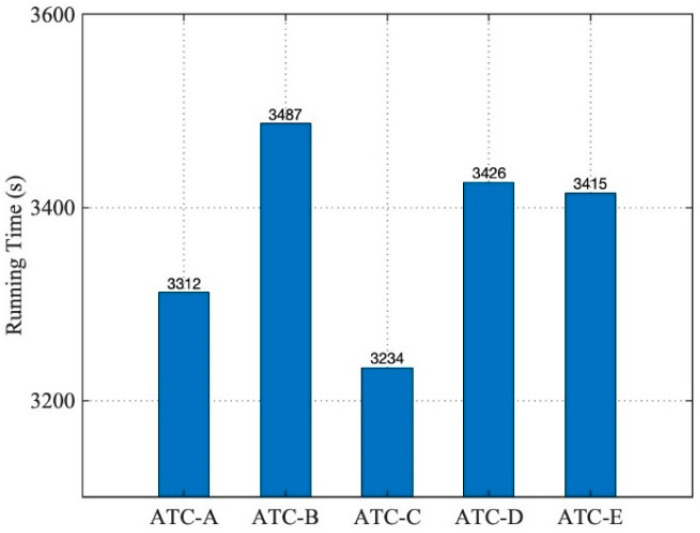
Running times of the five simulation systems (1).

The QoS weight vector gave a large weight to Q_M_, and the optimized candidate model set {ATC-C, ATC-A, ATC-E, ATC-D, ATC-B} could be obtained through calculation. The five ATC service models were assembled, respectively, to the same five airport operation control simulation systems, and the airliner instance was set to 100. The execution times of the five simulation applications operated by S-1P are shown in Figure 9.

These applications could run effectively, and it can be seen that in S-1P operation mode, the order of the ATC models corresponding to the execution efficiency of the five simulation applications was consistent with the optimization order in the model candidate set. The simulation application assembled by the model ATC-C, which ranked first in the candidate set, had the shortest running time (3234 s).

(2) In cloud architecture, shareable simulation services are often stored in the cloud center. The service model and simulation engine framework need to communicate through network interconnection. Both Q_C_ and Q_M_ of the simulation service model affect the execution efficiency of the simulation application, so the QoS preference weight vector was set to W = (0.35, 0.35, 0.1, 0.1, 0.1). The specific selection process is shown in Table 3.

**Figure 10:**
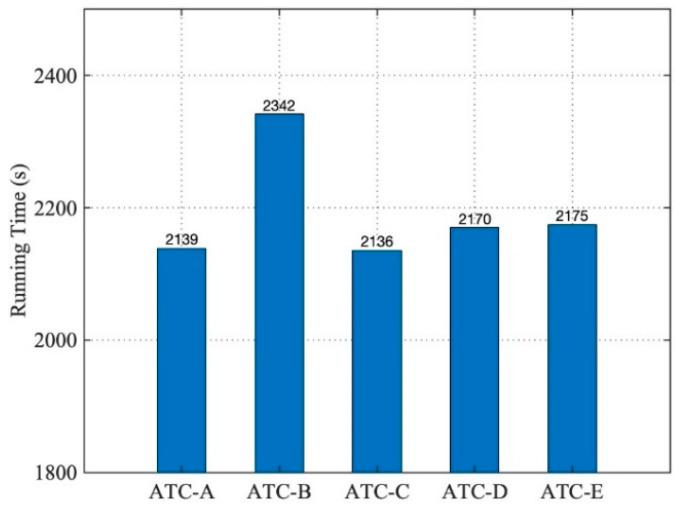
Running times of five simulation systems (2).

The QoS weight vector assigned the same weight to Q_C_ and Q_M_. After calculation, the optimized candidate model set could be obtained as {ATC-A, ATC-C, ATC-D, ATC-E, ATC-B}. The five ATC service models were assembled, respectively, to the same five airport operation control simulation systems, and the airliner instance was set to 100. The execution times of the five simulation applications operated by MS-CDS-2P are shown in Figure 10.

These applications could run effectively, and it can be seen that in MS-CDS-2P operation mode, the order of the ATC models corresponding to the execution efficiency of the five simulation applications was not completely consistent with the optimization order in the model candidate set. Because the QoS weight vector W was set merely according to the experimental architecture without detailed analysis, it was impossible to accurately quantify the extent to which the model performance Q_M_ and network communication capability Q_C_ affected the entire execution efficiency of the simulation application.

The experimental results show that the searched model can work together with other models and implement the simulation task, which verifies the effectiveness of the semantic search framework. Further, the proposed QoS-based simulation model selection method can support users in selecting the model which has the biggest target QoS index (Q) according to their QoS preference. However, it cannot always give the optimum solution that could optimize a certain performance (execution efficiency) of a complex simulation system.

### 5.2. Discussion

The experiment first tested the performance of the simulation application under three patterns: S-1P, TDS-2P, and CDS-2P. The results prove that, compared with TDS, CDS can also effectively improve the execution efficiency of the simulation application with little performance loss, which demonstrates the practicability of CDS. Experiment 2 encapsulated the models with large computational loads into shareable services in the cloud environment by the C-RUM specification proposed in this paper. Then, by comparing the performance of MS-CDS and Scatter-CDS, the results prove that the MS distribution mode is better than the traditional scatter distribution mode at improving the execution efficiency of complex system simulation. This demonstrates the feasibility of C-RUM specification in cloud networking architecture and the effectiveness of the method, making the models with large computational loads into shared services, proposed in this paper. In experiment 3, the required ATC models were found under the proposed semantic search framework by attributes or correlation searching in the model description knowledge graph. The experiment assembled the searched model into the simulation application of the case study and verified that it can work together with other models and effectively implement the simulation task, which verified the correctness of the semantic search framework. Then, the model ranking based on the QoS weighted selection method was compared with the ranking of the execution time of actual simulation systems assembled by the models in the candidate set. This proved that the proposed QoS weighted-based simulation model selection method can select simulation models according to users’ customized requirements, but the solution is not always the optimum one that could optimize the performance of the complex system simulation.

## 6. Summary and Future Work

A service-oriented model encapsulation and selection method for complex system simulation was proposed in this paper. This method first promotes the original RUM specification and puts forward C-RUM specification, which solves the problem of network communication in RUM specification. Models with large computational requirements are encapsulated into shareable services in the cloud architecture. The experimental results showed that model servitization can effectively improve the execution efficiency of complex system simulation applications. Then, the model semantic search framework is built in the simulation model description knowledge graph, which increases the correlation search ability compared with other semantic search methods. The QoS weighted-based model selection method supports users in customizing the weight of QoS indices and obtaining the ordered candidate model set by weighted comparison. This mechanism can support the selection of the required simulation model that satisfies users’ QoS preference under the cloud architecture.

Future work should further improve the QoS weighted-based simulation model selection method, considering the limitation that it cannot assist users in selecting the optimal simulation service model directed at a specific simulation application or its certain performance. Also, future research should confirm the metric of the model QoS indices and study how to assign the corresponding QoS index weights.

## Figures and Tables

**Table 1:** Experimental configuration.

Experimental Parameters	Description/Value
Number of airliners	(50, 250)
Number of airport runways	5
Number of air traffic control centers	1
Scheduling policy	Punctuality prioritized
Simulation run time	1008
Model distribution mode	Scatter, model servitization
Degree of parallelism	1, 2, 4

**Table 2:** Selection process 1.

	QoS1 [0,100]	QoS2 [0,100]	QoS3 [0,1]	QoS4 [0,1]	QoS5 [0,10]	Q
ATC-A	85 (0.85)	83 (0.83)	0.98	0.9	9 (0.9)	0.873
ATC-B	65 (0.65)	55 (0.55)	0.96	0.92	9 (0.9)	0.733
ATC-C	92 (0.92)	52 (0.52)	0.95	0.92	10 (1)	0.931
ATC-D	71 (0.71)	70 (0.7)	0.97	0.93	10 (1)	0.787
ATC-E	74 (0.74)	65 (0.65)	0.95	0.91	9 (0.9)	0.794
W=(0.7, 0, 0.1, 0.1, 0.1)						
Ordered candidate set: {ATC-C, ATC-A, ATC-E, ATC-D, ATC-B}						

**Table 3:** Selection process 2.

	QoS1 [0,100]	QoS2 [0,100]	QoS3 [0,1]	QoS4 [0,1]	QoS5 [0,10]	Q
ATC-A	85 (0.85)	83 (0.83)	0.98	0.9	9 (0.9)	0.866
ATC-B	65 (0.65)	55 (0.55)	0.96	0.92	9 (0.9)	0.698
ATC-C	92 (0.92)	52 (0.52)	0.95	0.92	10 (1)	0.847
ATC-D	71 (0.71)	70 (0.7)	0.97	0.93	10 (1)	0.7835
ATC-E	74 (0.74)	65 (0.65)	0.95	0.91	9 (0.9)	0.752
W=(0.35, 0.35, 0.1, 0.1, 0.1)						
Ordered candidate set: {ATC-A, ATC-C, ATC-D, ATC-E, ATC-B}						

## References

[1] Angelica S., Emanuele P., Andrea Z.P.S. (2018). The Role of Complex Analysis in Modelling Economic Growth. Entropy.

[2] Martin I., Jan C. (2017). A Behavioural Analysis of Complexity in Socio-Technical Systems under Tension Modelled by Petri Nets. Entropy.

[3] Yiping Y., Gang L. (2011). High-performance Simulation Computer for Large-scale System-of-Systems Simulation. Journal of System Simulation. J. Syst. Simul..

[4] Calheiros R.N., Ranjan R., Beloglazov A., Rose C.A.F.D., Buyya R. (2011). CloudSim: A toolkit for modelling and simulation of cloud computing environments and evaluation of resource provisioning algorithms. Softw. Pract. Exp..

[5] Simon J.E.T., Azam K., Katherine L.M., Andreas T., Levent Y., Justyna Z., Pieter J.M. (2015). Grand challenges for modelling and simulation: Simulation everywhere—from cyber infrastructure to clouds to citizens. Simulation.

[6] Feng Z., Yiping Y., Huilong C. (2014). Reusable Component Model Development Approach for Parallel and Distributed Simulation. Sci. World J..

[7] Sheng B., Zhang C. (2016). Common intelligent semantic matching engines of cloud manufacturing service based on OWL-S. Int. J. Adv. Manuf. Technol..

[8] Singh S., Chana I. (2015). QRSF: QoS-aware resource scheduling framework in cloud computing. J. Supercomput..

[9] Pujara J. (2013). Knowledge Graph Identification. Int. Semant. Web Conf..

[10] Yang S., Hanquan D., Minghua L. (2014). Research on Simulation Composability and Reusability Based on SOA. J. Syst. Simul..

[11] Lee H., Yang J.S., Kang K.C. (2014). Domain-oriented variability modeling for reuse of simulation models. Simulation.

[12] Jianbo L., Yiping Y. (2015). Research on the Development Approach for Reusable Model inParallel Discrete Event Simulation. Discrete Dyn. Nat. Soc..

[13] Yiping Y., Feng Z. (2013). A Reusable Simulation Model Development and Usage Method. China Patent.

[14] Purohit L., Kumar S. Web Service Selection using Semantic Matching. Proceedings of the International Conference on Advances in Information Communication Technology & Computing.

[15] Wu M.C., Gu J.Z. (2007). OWL-S Semantic Extension in the Dynamic Combination of Web services. Comput. Appl. Softw..

[16] Jiao H., Zhang J., Li J.H., Shi J. (2017). Research on cloud manufacturing service discovery based on latent semantic preference about OWL-S. Int. J. Comput. Integr. Manuf..

[17] Zhang T., Liu Y., Zha Y. (2007). Semantic Web-based approach to simulation services dynamic discovery. Comput. Eng. Appl..

[18] Song L.-L., Li Q. (2008). Research on simulation model description ontology and its matching model. Comput. Eng. Appl..

[19] Li T., Li B.H., Chai X.D. (2012). Layered simulation service description framework oriented to cloud simulation. Comput. Integr. Manuf. Syst..

[20] Cheng C., Chen A.Q. (2015). Study on Cloud Service Evaluation Index System Based on QoS. Appl. Mech. Mater..

[21] Tong Z., Yunsheng L., Yabing Z. (2009). Optimal Approach to QoS-Driven Simulation Services Composition. J. Syst. Simul..

[22] Jiahang L., Junli S., Liqun J. (2010). A QoS Evaluation Model for Cloud Computing. Comput. Knowl. Technol..

[23] Li B.H., Chai X., Hou B., Li T., Zhang Y.B., Yu H.Y., Tang Z. (2009). Networked Modeling & Simulation Platform Based on Concept of Cloud Computing—Cloud Simulation Platform. J. Syst. Simul..

[24] Chen T., Chiu M.C. (2017). Development of a cloud-based factory simulation system for enabling ubiquitous factory simulation. Robot. Comput. Integr. Manuf..

[25] Jeanjacques M.C. (2003). Web services Description Language (WSDL) Version 1.2.

[26] Xu Z.L., Sheng Y.P., He L.R., Wang Y.F. (2016). Review on Knowledge Graph Techniques. J. Univ. Electron. Sci. Technol. China.

[27] Organization T.M., Shekar S., Xiong H. (2004). Resource Description Framework (RDF). Encyclopedia of Gis.

[28] Siqi X., Feng Z., Yiping Y., WenJie T. A Description Method of Cloud Simulation Model Resources based on Knowledge Graph. Proceedings of the 4th International Conference on Cloud Computing and Big Data Analytics, IEEE.

[29] You M., Wang S., Hung P.C.K. (2017). A Highly Accurate Prediction Algorithm for Unknown Web Service QoS Values. IEEE Trans. Serv. Comput..

[30] Francis N., Green A., Guagliardo P. (2018). Formal Semantics of the Language Cypher. arXiv.

